# Incorporating causal inference perspectives into psychoneuroimmunology: A simulation study highlighting concerns about controlling for adiposity in immunopsychiatry

**DOI:** 10.1016/j.bbi.2023.06.022

**Published:** 2023-06-29

**Authors:** Daniel P. Moriarity, Summer Mengelkoch, George M. Slavich

**Affiliations:** Department of Psychiatry and Biobehavioral Sciences, University of California, Los Angeles, CA, USA

**Keywords:** Inflammation, Depression, Adiposity, BMI, Causal inference

## Abstract

Psychoneuroimmunology and immunopsychiatry are quickly approaching a critical point where the clinical translatability of their evidence base will be tested. To maximize chances for translational success, we believe researchers must adopt causal inference techniques that augment the causal relevance of estimates given theorized causal structures. To illustrate the utility of incorporating causal inference perspectives into psychoneuroimmunology, we applied directed acyclic graphs and a combination of empirical and simulated data to demonstrate the consequences of controlling for adiposity when testing the association between inflammation and depression under the plausible causal structure of increases in adipose tissue leading to greater inflammation that in turn promotes depression. Effect size estimates were pulled from a dataset combining the Midlife in the United States 2 (MIDUS-2) and MIDUS Refresher datasets. Data were extracted and used to simulate data reflecting an adiposity → inflammation → depression causal structure. Next, a Monte Carlo simulation study with 1,000 iterations and three sample size scenarios (*N*s = 100, 250, and 500) was conducted testing whether controlling for adiposity when estimating the relation between inflammation and depression influenced the precision of this estimate. Across all simulation scenarios, controlling for adiposity reduced precision of the inflammation → depression estimate, suggesting that researchers primarily interested in quantifying inflammation → depression associations should not control for adiposity. This work thus underscores the importance of incorporating causal inference approaches into psychoneuroimmunological research.

## 1. Introduction

Over the past 50 years, the field of psychoneuroimmunology has yielded substantial evidence illustrating how human thoughts, feelings, and behaviors affect the immune system and vice versa. With this knowledge in hand, a primary focus for the next era will be to translate this basic science of psychoneuroimmunology into clinical interventions aimed at improving immune-mediated somatic and psychiatric health outcomes. Success in this mission, we believe, will be determined by the field’s willingness to adopt methodological techniques and perspectives that maximize the causal inferences that can be gleaned from psychoneuroimmunological studies.

Causal inference is an entire field of scientific inquiry dedicated to estimating the magnitude of causal effects given theorized causal structures (also described as “data generating mechanisms”). Specifically, beyond experimental designs, causal inference principles describe the conditions required to maximize the causal implications of observational data [see Morgan and Winship (2015) for an overview specific to observational social science data]. The primary aim of this article is to illustrate the consequences of observational psychoneuroimmunological research that does not abide by these principles using a combination of empirical data and Monte Carlo simulations. To accomplish this aim, we first provide a brief introduction to core benefits of causal inference perspectives and discuss how they maximize the causal relevance of research. Second, we use the common decision to control for adiposity/body mass index (BMI) when looking at inflammatory predictors of depression as a widely relevant use-case to demonstrate how this common strategy may reduce the precision of key effect estimates in immunopsychiatry. Finally, we conclude with recommendations for psychoneuroimmunologists looking to leverage the field of causal inference to improve their own work.

### 1.1. A Primer on Causal Inference

A complete review of the field of causal inference can be found elsewhere, such as in Dablander’s didactic introduction to causal inference (Dablander, 2020). In brief, causal inference methodologies improve the causal relevance of research by (a) establishing a formal causal hierarchy, (b) accounting for bias (including bias that cannot be addressed using statistical control), and (c) improving the precision of effect estimates (Pearl and Machenzie, 2018). Below, we briefly introduce the causal hierarchy and implications for bias, as implications for precision will be the focus of this article and will be discussed in greater detail below.

The causal hierarchy is typically described as having three levels: association, intervention, and counterfactuals (Pearl and Machenzie, 2018; Pearl, 2019). These levels are listed in order of both (a) least-to-most causally relevant and (b) least-to-most intensive assumptions. These levels are also referred to as their three associated actions: seeing, doing, and imagining, respectively. Association/seeing refers to studies using observational data, which will be the level relevant to our use case below (i.e., what is the association between inflammation and depression symptoms). Although the use of causal inference methods such as directed acyclic graphs (described below) does not enable researchers to make causal claims from observational data, considering causal associations between variables of interest can reduce bias and improve estimate precision by informing study design, data collection, and analysis (Pearl, 1995). The second level of the hierarchy, intervention/doing, involves manipulation of variables to answer population-level questions (e.g., what would happen if all patients with depression were forced to take an anti-inflammatory medication?). The final level of the hierarchy is counterfactuals/imagining, which enables exploration of individual-level questions (e.g., would patient X have recovered if they had received this medication, even though they neither recovered nor received this medication in reality).

In well-intentioned efforts to increase the causal relevance of observational data, many researchers decide to statistically control for potentially influential “third variables”. Unfortunately, it is not uncommon for this to result in a “kitchen sink”/“garbage can” approach of controlling for all variables that are potentially related to the focal predictor and outcome (Achen, 2005) without considering the underlying causal relations between the variables and their implications for bias and precision. Directed acyclic graphs (DAGs) are a common tool in causal inference used to formalize assumptions about these causal relations to inform modeling decisions. For example, the use of DAGs can help avoid certain systematic sources of bias, such as confounding bias. A confounder is, by definition, a third variable that has a causal influence on two other variables that might be associated. Controlling for a confounder removes this bias.

**Fig. 1.**
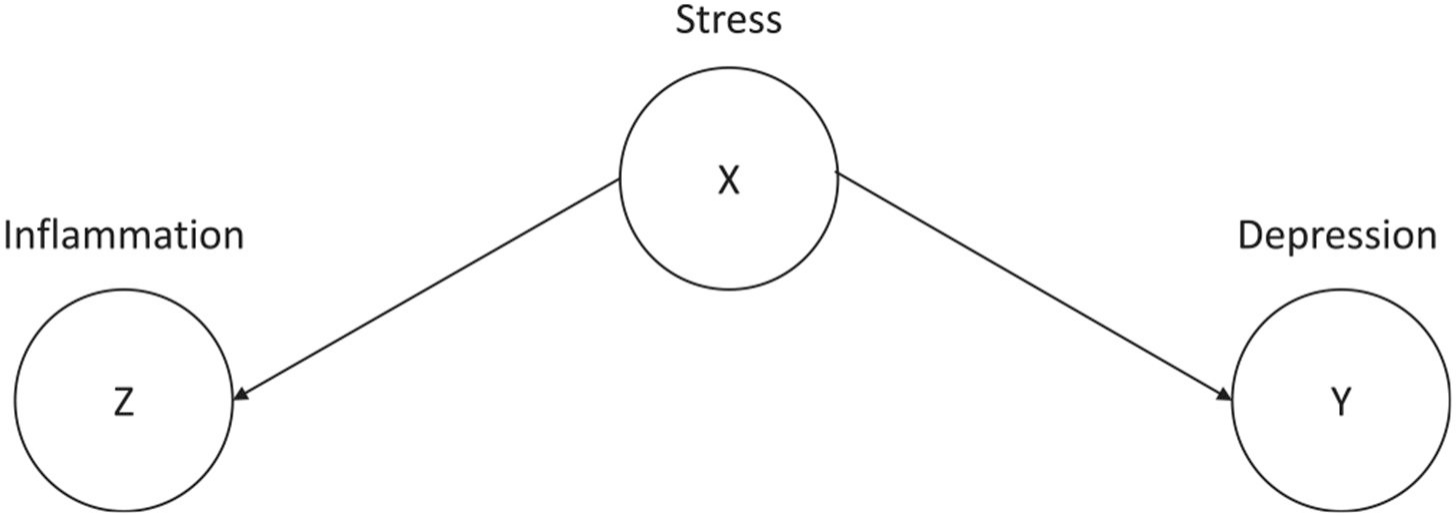
Example Directed Acyclic Graph – Stress as a Confounder.

**Fig. 2.**
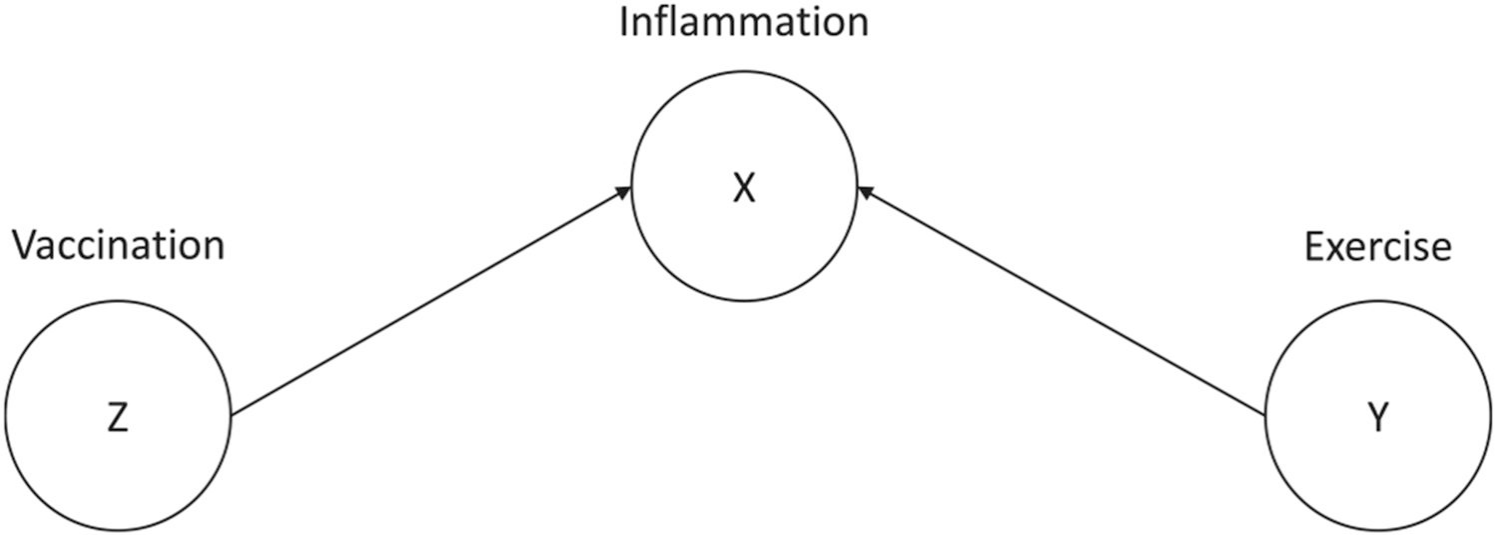
Example Directed Acyclic Graph – Inflammation as a Collider.

Specific to psychoneuroimmunology, acute stress can be thought of as a confounder of the relation between inflammatory biology and depression (for an example DAG, see Fig. 1). A second type of bias that can be identified using DAGs is collider bias. Collider bias arises when controlling for a variable that is caused by the two variables of interest, which can cause spurious correlations between the two variables and should be avoided (Rubin, 1974; Rosenbaum, 1984; Elwert and Winship, 2014). For example, both receiving a vaccination and recent exercise can increase inflammation, but there is plausibly no causal relation between vaccination and recent exercise (Fig. 2). In this scenario, controlling for inflammation would be conditioning on a collider—potentially biasing the association between vaccination and recent exercise.

Here, we briefly describe the key assumptions for DAGs [see Rohrer (2018) for a detailed description of DAGs]. Simply put, DAGs are visual tools used to specify causal assumptions using nodes (variables) and edges (arrows) that reflect causal relations (Pearl, 1995). One of the core assumptions of DAGs is that direct manipulation of one node will cause changes in downstream nodes. Importantly, DAGs only allow for singleheaded arrows (i.e., no bidirectional relations) because no nodes are allowed to proceed themselves—described as a “cyclic” association. However, separate DAGs can be constructed for different measurement occasions to incorporate concepts of A → B and, later, B → A. Under the right conditions, the use of Structural Causal Models can be used to formalize bidirectional associations. Structural Causal Models incorporate all three levels of causality and translate causal statements to probabilistic statements that can be empirically tested.

**Fig. 3.**
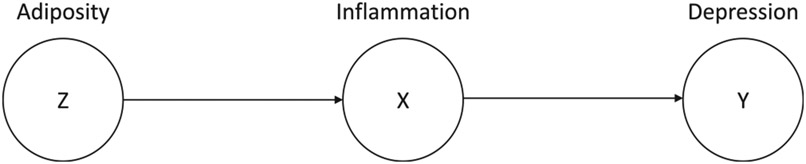
Example Directed Acyclic Graph – Adiposity, Inflammation, and Depression.

Although more complex causal structures exist, all can be broken down into three fundamental causal structures: chains, forks, and inverted forks (Elwert, 2013). Chains represent a causal path from A → B → C (e.g., Fig. 3). Forks describe A ← B → C paths (see Fig. 1), whereas inverted forks describe A → B ← C paths (see Fig. 2). In these paths, nodes at the receiving end of edges are referred to as “descendants” whereas nodes at the beginning of edges are referred to as “ancestors”. Any path that contains an inverted fork is “blocked” because the associations cannot be transmitted behind the ancestor node. Using DAGs to map out these associations can inform analytic decisions to reduce bias (described above) as well as maximize precision of effect estimates.

### 1.2. Precision Amplifies Translational Impact

As the focus of psychoneuroimmunology research shifts toward translational impact, the ability to precisely quantify *the size* of effects between psychology, behavior, and immunology is equally as important as the ability to detect *if* an effect exists. Consider the development of a randomized clinical trial testing immune-modulating medications as an augmentative treatment to a psychosocial intervention for depression. The immune system is an immensely diverse, multifaceted biological system that presents a variety of possible treatment targets (Capuron and Miller, 2004; Raison et al., 2006, 2013; Felger and Miller, 2020; Nettis et al., 2021). Selecting the most promising immune-modulating medications requires a comparison of effect sizes between candidate treatment targets (i.e., proteins with an identified association) and depression. Imprecise effect size estimates decrease clarity of this choice and could lead to investment in suboptimal treatments, resulting in inefficiently stewarded resources (e.g., research funding, time) and limited clinical impact. The same rationale applies to selecting behavioral interventions to alter immunology. Solely understanding that behaviors, such as dietary routines and exercise, influence immune functioning is not enough to determine the optimal, first-line behavioral intervention for rheumatoid arthritis; rather, it is necessary to accurately quantify and compare the effects of specific diet changes and exercise programs (amongst other interventions) to optimize treatment recommendations. Further, imprecise effect size estimates could result in underpowered clinical trials, further slowing translation of basic psychoneuroimmunology into real world impact.

### 1.3. Plausible Causal Structure of Adiposity, Inflammation, and Depression

To further illustrate the nature of these issues, we turn now to a specific example: controlling for adiposity when investigating immunological associations with depression. Controlling for adiposity or a proxy for adiposity, such as BMI, is common practice in immunopsychiatry, and is formally recommended in some widely cited articles on covariates in psychoneuroimmunology (O’Connor et al., 2009; Horn et al., 2018). The primary rationale for doing this is that many commonly studied inflammatory proteins are created and released by macrophages and adipocytes in fat tissue (Shelton and Miller, 2011; Ellulu et al., 2017). For example, approximately 30% of circulating interleukin (IL)-6 is estimated to originate from adipose tissue (Mohamed-Ali et al., 1998). This hypothesis has also inspired research examining the extent to which inflammation may mediate the association between adipose tissue and depression (visualized in Fig. 3; Capuron et al., 2011; Shelton and Miller, 2011; Daly, 2013; Chu et al., 2023). Although we did not find any studies that tested true mediation models of these associations using repeated measures of all focal variables to assess change (i.e., changes in predictor → changes in mediator → changes in outcome), this is a compelling causal framework. Indeed, this causal model is often the primary rationale for the widespread acceptance of adiposity/BMI as a recommended covariate when testing immunology as a predictor of psychopathology and behavioral outcomes (O’Connor et al., 2009; Horn et al., 2018). As highlighted above, however, this rationale does not classify adiposity as a confounder of the inflammation → depression association. In fact, the application of causal inference principles highlights that this well-intentioned standard might have negative consequences for statistical model performance.

In Fig. 3, we use a DAG to illustrate a causal chain wherein adiposity affects depression through inflammation, which has been proposed by several theories of depression (Slavich and Irwin, 2014; Miller et al., 2009). Critically, there are a variety of established implications of controlling for Z in different causal structures when the association between X and Y is the effect of interest [for a more thorough primer on how to use DAGs to determine the consequences of covariate selection, see Supplement 3 of Del Giudice and Gangestad (2021)]. As we will illustrate in the present study (below), controlling for Z in the scenario depicted in Fig. 3 will invariably decrease measurement precision of the X →Y estimate, when there is no direct effect of adiposity above and beyond the indirect effect via inflammation.

## 2. The Present Study

The present study uses both empirical data and simulations to illustrate the consequences of controlling for a more distal causal variable when trying to quantify the relation between a downstream variable (i.e., a mediator) and the outcome when the distal variable has no direct effect itself on the outcome. Specifically, we leverage the decision to control for adiposity when quantifying the inflammation → depression association as a widely-relevant use case. Effect sizes are identified using two open datasets (described below). These effect sizes are then used to simulate datasets to compare the precision of inflammation → depression estimates with and without controlling for adiposity to a known, “true” effect size.

## 3. Method

### 3.1. Participants and Procedure

Effect sizes for the simulation were extracted from a dataset combining data from the Midlife in the United States 2 [MIDUS-2; Ryff et al. (2017)] and MIDUS Refresher (MIDUS-R; Weinstein et al. (2017)] datasets. MIDUS-2 studied 1,255 (*M*_*age*_ = 55.42 years, 50% female, 78% White) participants aged 25-75 years old who were fluent in English and volunteered to participate in a biomarker collection protocol that included a sera assessment of eight inflammatory proteins [i.e., C-reactive protein (CRP), IL-6, IL-8, IL-10, tumor necrosis factor-α (TNF-α), fibrinogen, E-selectin, and intercellular adhesion molecule-1 (ICAM-1)]. Beyond fibrinogen, all of these proteins are found in adipose tissue. Therefore, all proteins except fibrinogen were tested. MIDUS-R was designed to parallel MIDUS-2’s methodology and consisted of 863 adults (*M*_*age*_ = 53.53 years, 50% female, 87% White). After combining datasets and removing missing data (various participants in MIDUS had adiposity quantified using different methods, described below), the analytic sample size for the mediation analyses ranged from 543-549 depending on the protein tested.

### 3.2. Measures

#### Total Body Adiposity.

Total grams of whole body fat mass was measured using Lunar DXA scanners. Different MIDUS data collection sites used different scanners, and our decision to use the Lunar scanner data maximized our analytic sample size as it was the only adiposity measurement included in both MIDUS-2 and MIDUS-R datasets.

#### Inflammatory Proteins.

Fasting blood draws were collected between 6:00 and 8:30 am for both studies. Blood was centrifuged and plasma and serum samples were stored in a −60 to −80° C freezer. Samples were shipped to the MIDUS Biocore Lab on dry ice, where they were stored at −65 °C until assayed. CRP originally was analyzed in plasma via BNII nephelometer (Dade Behring Inc.). Samples falling below the assay range for this method were re-assayed using immunoelectrochemiluminescence using a high-sensitivity assay kit [Meso Scale Diagnostics (MSD)]. Beginning in 2016, all participants (*n* = 150 from MIDUS-R) had CRP assayed using the MSD platform using serum. Corrections to account for these changes were applied before the data were made publicly available and analyzed for this study. E-Selectin and ICAM-1 were measured using enzyme-linked immunosorbent assays (ELISAs; R&D Systems, Minneapolis, MN). Lot-to-lot changes in both E-Selectin and ICAM-1 assays were made throughout the course of the study and adjusted for prior to the data being made publicly available. Cytokines (IL-6, IL-8, IL-10, and TNF-α) were quantified by V-plex Custom Human Cytokine Kit (MSD, Rockville, MD), MSD Sulfo-tag, and MSD Sector Imager. E-Selectin and ICAM-1 values outside of the detectable range (LLOD = <.1 ng/mL and <45 mg/L, respectively) were set at .09 ng/mL and 44 ng/mL, respectively. MIDUS documentation (Ryff et al., 2017; Weinstein et al., 2017) indicates that none of the other proteins had values outside of the detectable range. Assay ranges and variability for all proteins can be found in the MIDUS documentation available online (Ryff et al., 2017; Weinstein et al., 2017).

#### Depression Symptoms.

The Center for Epidemiological Studies Depression Inventory (CES-D) quantified depression symptoms. The CES-D is a 20-item questionnaire with a 4-point Likert response scale. Scale scores were summed by computing the sum of all items for observations in which there was no missing data, after reverse scoring appropriate items (Cronbach’s α = .89).

### 3.3. Effect Size Extraction

Unfortunately, none of the studies we found that tested inflammation as a mediator of the association between adiposity or adiposity proxies (e.g., BMI) and depression [e.g., Capuron et al. (2011); Daly (2013); Chu et al. (2023)], tested repeated measures of all focal variables (i.e., changes in adiposity → changes in inflammation → changes in depression) and reported standardized effect sizes that could be used for simulating the data necessary for this study. To acquire standardized effect size estimates, MIDUS-2 and MIDUS-R datasets were combined and analyzed using Model 4 of the PROCESS V4.2 SPSS macro (Hayes, 2017), resulting in analytic sample sizes of 543-549 for the mediation analyses, depending on the protein tested. Each of the seven proteins was tested as a mediator of the association between total body adiposity and depression. Protein values were not transformed because the assumption of normality in linear models refers to the residuals (the PROCESS macro does not give this diagnostic) not the normality of the values (Ernst and Albers, 2017) as commonly reported in psychoneuroimmunology research (Moriarity, 2022).

### 3.4. Monte Carlo Simulation

The Monte Carlo simulation study was conducted in R Version 4.2.2 (Team RC, 2013). Briefly, a Monte Carlo simulation involves simulating multiple datasets with “known” effects between the variables of interest. Because the data were generated with the effect sizes specified, it is already known what the “true” answer is when trying to quantify these effects (e.g., the association between inflammation and depression). This gold standard can then be used as a benchmark to compare analyses run on the simulated data to determine which of a set of analytic approaches (e.g., regressions with inflammation predicting depression with, and without, controlling for adiposity) is most effective at quantifying or detecting the “true” effect size.

The effect sizes observed in the MIDUS data were used to simulate a dataset reflecting a causal association from adiposity, through an inflammatory protein, to depression. Data were managed using tidyverse (Wickham et al., 2019). Data were simulated using *lavaan* (Rosseel, 2012). Simulation results were estimated and visualized using *rsimsum* (Gasparini, 2018). Three Monte Carlo simulations with 1,000 different samples were conducted. The three simulation scenarios only differed in the size of each sample (*N*s = 100, 250, and 500) to illustrate that larger sample sizes do not change our conclusions. Data were simulated to reflect a causal structure where an inflammatory protein mediated the association between adiposity and depression according to the proxy effect sizes found in the cross-sectional mediation analyses. Two regressions were estimated in each simulated dataset: one with inflammation predicting depression and one with inflammation predicting depression while controlling for adiposity. Each estimate of the association between inflammation and depression was extracted and compared to the “true” effect size (i.e., the effect size that the simulated data was based on) between inflammation and depression to compare model performance. The key metric of interest is the precision of the model estimates. The code for the data simulation and simulation performance analyses are available in the Supplemental Materials. A correlation matrix of MIDUS variables can be found in Supplemental Table 1**.**

## 4. Results

### 4.1. Effect Size Extraction

**Fig. 4.**
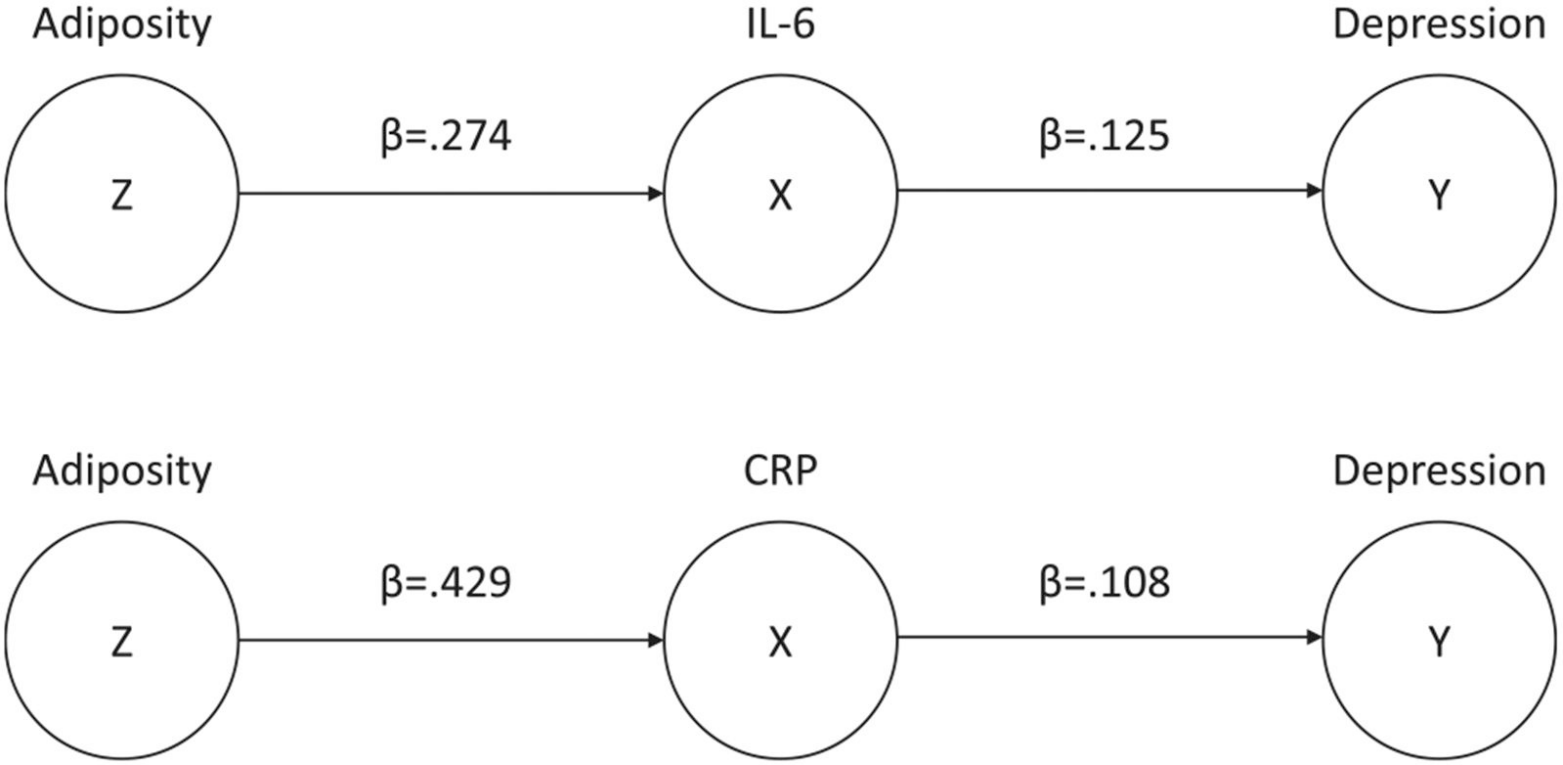
Directed Acyclic Graphs for Simulations.

Of the proteins assessed, IL-6 and CRP were the only proteins that accounted for a significant indirect effect of adiposity on depression symptoms (IL-6: β = .034, bootstrapped 95% CI [.015, .070]; CRP: β = .046, bootstrapped 95% CI [.008, .086]). After accounting for these indirect effects, the direct effect of adiposity on depression symptoms was null in both models (IL-6: *p* = .193; CRP: *p* = .376), consistent with some prior longitudinal research on inflammatory proteins as a mediator of the association between excess body weight and somatic depression symptoms (Chu et al., 2023). The a’ and b’ pathways were significant in both models, with more adiposity predicting higher inflammatory protein levels (IL-6: β = .274, *p* < .001; CRP: β = .429, *p* < .001), and higher inflammatory protein levels predicting more depression symptoms (IL-6: β = .125, *p* < .001; CRP: β = .108, *p* = .023), respectively. See Fig. 4 for the resulting DAGs.

### 4.2. Monte Carlo Simulation

**Fig. 5.**
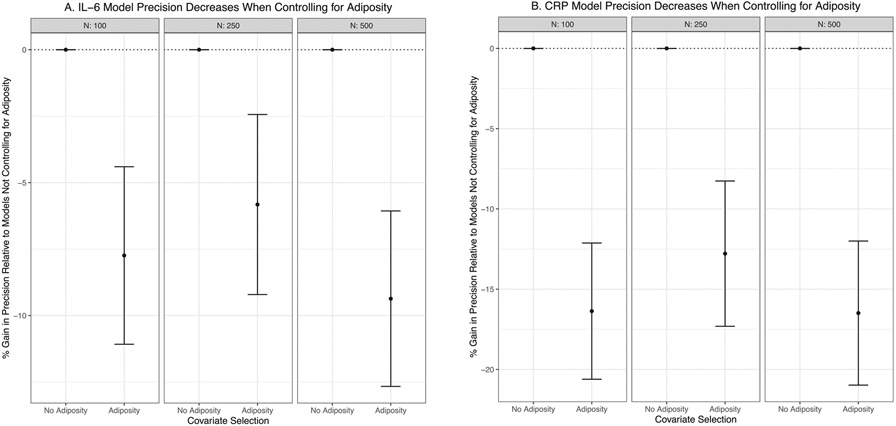
Precision Decreases when Controlling for Adiposity. Models with inflammation predicting depression without adiposity as a covariate are used as a reference group. Consequently, the “No Adiposity” condition is always at 0% relative gain in precision because it is compared to itself. Controlling for adiposity resulted in a loss of precision for all sample sizes. Confidence intervals are based on Monte Carlo standard errors.

**Fig. 6.**
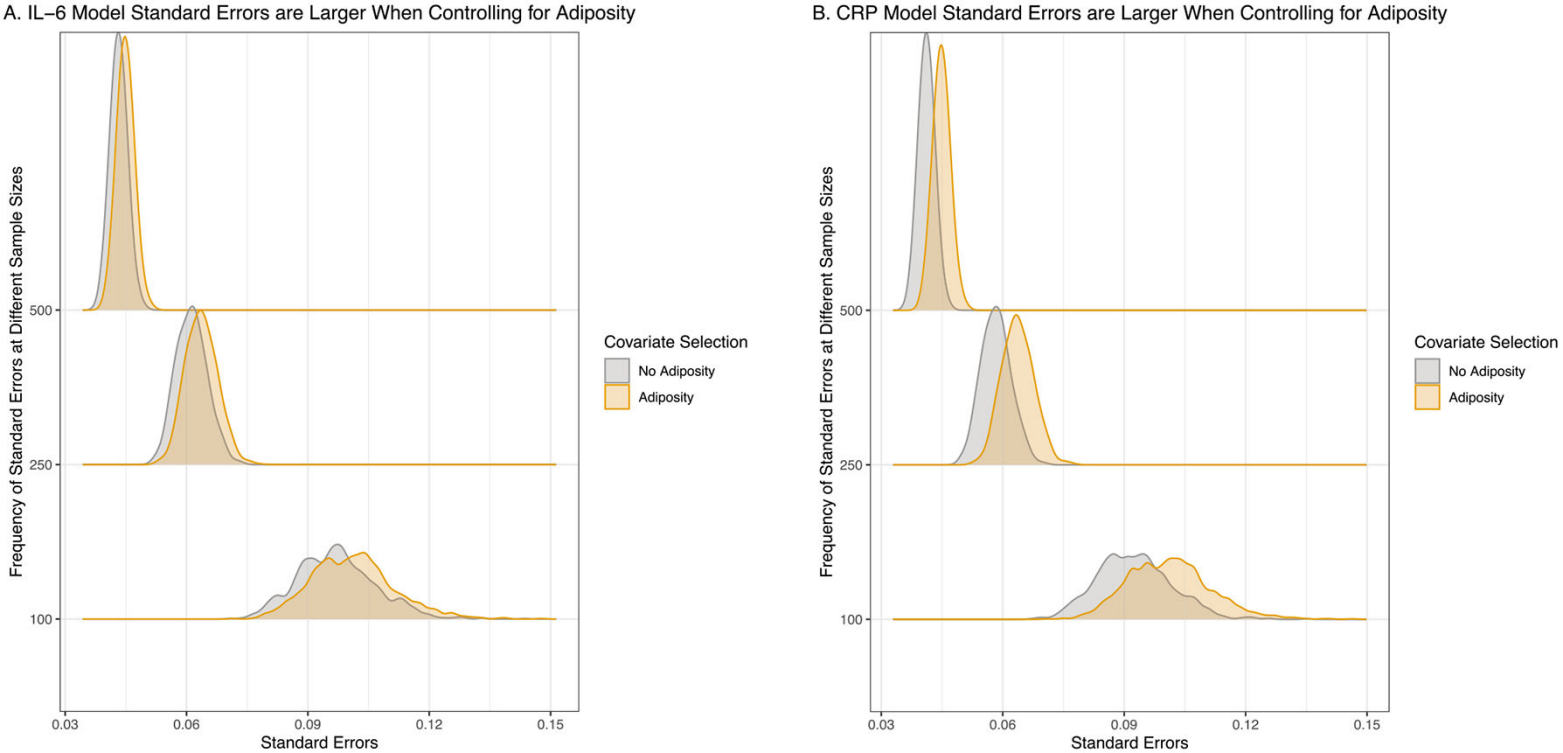
Standard Errors are Larger When Controlling for Adiposity. Ridge plots of the distributions of the standard errors for each sample size condition. As would be expected, standard errors increased as the sample sizes decreased. Additionally, in all three scenarios, controlling for adiposity resulted in higher standard errors compared to not controlling for adiposity, as indicated by the yellow distributions being shifted farther to the right relative to the grey distributions

As hypothesized, the observed effect size between both inflammatory proteins and depression was less precise in models controlling for adiposity, regardless of sample size. Specifically, effect estimates for the IL-6 models were 7.7%, 5.8%, and 9.4% less precise, and the estimates in the CRP models were 16.4%, 12.8%, and 16.5% less precise (for the simulations with *N* = 100, *N* = 250, and *N* = 500, respectively) in models covarying for adiposity relative to models not covarying for adiposity (Fig. 5). Relatedly, standard errors for the inflammation →depression effects were larger for models controlling for adiposity in all three sample size scenarios (Fig. 6) for both the IL-6 (*N* = 100: *t* = −9.08, *p* < .001; *N* = 250: *t* = −13.94, *p*< .001; *N* = 500: *t* = −18.58, *p* < .001) and CRP models (*N* = 100: *t* = −19.78, *p* < .001; *N* = 250: *t* = 31.41, *p*< .001; *N* = 500: *t* = −43.39, *p* < .001). Also of interest, power was higher in all three scenarios when adiposity was not modeled as a covariate for both the IL-6 (*N* = 100: no adiposity = 29% power vs. control for adiposity = 28% power; *N* = 250: no adiposity = 57% power vs. control for adiposity = 52% power; *N* = 500: no adiposity = 87% power vs. control for adiposity = 84% power) and CRP models (*N* = 100: no adiposity = 28% power vs. control for adiposity = 25% power; *N* = 250: no adiposity = 53% power vs. control for adiposity = 46% power; *N* = 500: no adiposity = 80% power vs. control for adiposity = 73% power).

## 5. Discussion

As psychoneuroimmunology attempts to realize its potential for clinical translation, it will be critical for data to be collected and analyzed in ways that maximize the causal inferences that can be made from the studies conducted. To illustrate this important point, we applied concepts from the field of causal inference to demonstrate how a common recommendation in psychoneuroimmunology (O’Connor et al., 2009) — to control for adiposity when quantifying the association between inflammation and depression — might systematically reduce precision of inflammation → depression estimates.

Leveraging the power of Monte Carlo simulations and open datasets (MIDUS-2 and MIDUS-R), we simulated data to reflect a causal structure wherein inflammation mediates the relation between adiposity and depression, a common rationale for controlling for adiposity when testing inflammatory predictors of mental health and inspiration for several mediation studies and perspective pieces (Shelton and Miller, 2011; Daly, 2013; Chu et al., 2023). By creating a DAG to illustrate this causal structure, we were able to refer to the causal inference literature on whether controlling for adiposity would improve or impair a statistical model’s ability to accurately quantify the inflammation → depression association. Consistent with recommendations from the causal inference literature to avoid selecting covariates that primarily influence the outcome by its downstream effect on the focal predictor (Del Giudice and Gangestad, 2021), our simulations demonstrated that controlling for adiposity reduced the precision of inflammation → depression estimates for two different proteins. However, it is important to note that this finding would be less straightforward if adiposity had a significant direct effect on depression above and beyond the indirect effect via inflammation [which was the case in Daly (2013) but not in the MIDUS data used here or in Chu et al. (2023)]. In this situation, the DAG in Fig. 3 should be revisited and the costs/benefits of controlling for adiposity reconsidered. Specifically, it might be more appropriate to control for adiposity if the indirect effect of adiposity on depression via inflammation is substantially smaller than the direct effect of adiposity on depression, in which case adiposity would primarily function as a confounder of the inflammation → depression association.

Imprecise estimation of effect sizes is a meaningful hurdle that must be overcome to effectively prioritize treatment targets, design clinical trials, and shorten the research-to-clinical impact pipeline. Even relatively small drops in precision induced by controlling for adiposity (in this example) are important given that this is just one of many potential sources of precision reduction and/or bias in the many choices researchers make [e.g., other covariates, measurement error in the variables, non-optimal time lags (Dormann and Griffin, 2015), improper choice of statistical models]. Further, given that inflammation → depression effect sizes are generally small, increases in standard errors (as observed here) can have substantial impact on both standardized effect sizes and *p*-values. Because the immune system is host to dozens of potential treatment targets, it is imperative that researchers designing clinical trials can compare effect sizes from pre-clinical studies to identify ideal treatment targets. Consider the hypothetical example of a research team designing a clinical trial for an immune-modulating augment to a psychosocial intervention for depression. Before selecting what augmentative treatments to consider, it is necessary to decide which immune mechanism to target. If the researchers have slimmed their options to IL-6 or CRP, they would want to decide which protein to target based on which protein is most strongly associated with depression. Consider that both proteins are tested as predictors of depression symptoms while controlling for adiposity, resulting in 9.4% and 16.5% drops in precision, respectively. If the effect size of IL-6 is underestimated by 9.4% and the effect size of CRP is overestimated by 16.5%, this equates to a “swing” of 25.9% and could misinform protein-level targets for subsequent intervention trials. Only if effect sizes are precisely estimated can they be meaningfully compared to select the immune target(s) and intervention strategies that will provide the greatest return on investment for grant funding agencies, research teams, and the patients we serve.

### 5.1. Considering Cyclic Relations and Moderators in DAGs

Readers may note that many processes of interest to psychoneuroimmunology researchers are often complex, characterized by bidirectional associations and/or influenced by moderators, and wonder how these nuances might influence the use of causal inference tools or the recommendations in this article. First, we discuss cyclic/bidirectional pathways. In addition to adiposity being a source of inflammatory proteins, there is some evidence to suggest that chronic inflammation can itself promote adiposity via impaired leptin response (Pérez-Pérez et al., 2020), implicating a potential inflammation → adiposity → more inflammation → depression causal chain. In the case that inflammation → depression is the primary association of interest, the recommendation would still be to avoid controlling for adiposity both for the rationale illustrated in the current study (i.e., controlling for adiposity will decrease precision of the estimate of the inflammation → depression effect as long as there is no direct effect of adiposity on depression) but also because controlling for a mediator, by definition, will bias the observable effect size between a predictor and an outcome to underestimate this association. There is also support for bidirectional associations between inflammation and depression (Moriarity et al., 2020). Cyclic/bidirectional processes cannot be included in singular DAGs, they have to be specified in separate DAGs associated with disparate timepoints (e.g., T1 DAG inflammation → depression, T2 DAG depression → inflammation). This process is valid because, by their nature, cyclic associations require more time to unfold. Therefore, at a small enough time scale, all causal effects are acyclic. Alternatively, under certain conditions, Structural Causal Models can also model cyclic relations (resulting in directed cyclic graphs). Finally, there are many potential moderators in PNI [e.g., sex (Alanna et al., 2011; Moieni et al., 2015; Moriarity et al., 2019)]. Traditionally, DAGs are nonparametric and, therefore, whether ancestors/causal determinants interact which each other is irrelevant. However, interaction DAGs (IDAGs; Nilsson et al., 2021) do exist in which, instead of nodes for an outcome, there are nodes for causal effects. IDAGs extend the concepts of DAGs to different types of interactions (e.g., confounded interactions, total vs. direct vs. indirect interactions), which allow for causal mapping of the complex moderating relations often studied by psychoneuroimmunologists.

### 5.2. Recommendations for the Field

Looking forward, there are several recommendations psychoneuroimmunology researchers can adopt to ensure that their work (observational or otherwise) maximizes its causal, and consequently clinical, relevance. These recommendations are summarized in Table 1. First, we encourage readers to familiarize themselves with the foundational perspectives and tools of causal inference. Specifically, we recommend the introduction in Dablander (2020) or its associated blog post for a more digestible introduction to causal inference (see https://fabiandablander.com/r/Causal-Inference.html). Second, we suggest that researchers deeply consider the plausible causal structure of their variables of interest, including covariates (Rohrer, 2018; Wysocki et al., 2022). This process should include reflection on longitudinal and experimental research regarding the temporal ordering of causal effects between variables. Even in studies where researchers have no intention to claim causal effects, formalizing the theorized causal structure using DAGs can inform a variety of different analytic decisions to improve causal relevance. For example, in this study, we illustrated how controlling for a third variable (i.e., adiposity) that has a causal effect on a focal predictor (i.e., IL-6 or CRP), reduces estimate precision when quantifying associations between the focal predictor and downstream descendants (i.e., depression symptoms). Third, we discourage the use of the “kitchen sink” method of covariate selection occasionally embraced by researchers attempting to be as conservative as possible (Achen, 2005). Although well-intentioned, selecting covariates without careful consideration of causal relations can obscure meaningful effects and reduce the estimate precision of key associations between immunology and psychology. As illustrated here, and described in further detail in Rohrer (2018), Del Giudice and Gangestad (2021), and Wysocki et al. (2022), this can negatively impact the quality of the study and delay the advancement of psychoneuroimmunology. Finally, we believe it is important for all studies in psychoneuroimmunology to report standardized effect size estimates. Although it would not have changed the pattern of results (as long as there was no main effect of adiposity on depression above and beyond its indirect effect via inflammation), this simulation study would ideally have used standardized effect sizes from published longitudinal research. Unfortunately, the longitudinal mediation studies we found (Daly, 2013; Chu et al., 2023) only reported unstandardized effect sizes, resulting in this study having to use cross-sectional data to estimate proxy effect sizes. Further, consistent reporting of standardized effect sizes is integral to empowering those developing interventions to select the most promising treatment targets and strategies.

## 6. Conclusion

In closing, psychoneuroimmunologists are inherently interdisciplinary and, as such, are well-poised to embrace the methodological techniques and perspectives of causal inference. By applying these techniques and perspectives, both when designing studies and when interpreting results, psychoneuroimmunology researchers will be able to tighten the gap between basic science and clinical impact, maximizing the effectiveness of interventions that have the potential to improve immune-mediated somatic and psychiatric health outcomes.

## Supplementary Material

supplemental code crp

supplemental code il6

supplemental data

supplemental table 1

## Figures and Tables

**Table 1:** Recommendations for Psychoneuroimmunologists.

Recommendations		Implementation
1.	Familiarize yourself with the foundational perspectives and tools of causal inference	See Dablander (2020) for an introduction to causal inference or its associated blog post: https://fabiandablander.com/r/Causal-Inference.html.
2.	Carefully consider the causal structure of variables of interest, including covariates	Formalize the theorized causal structure of your variables using Directed Acyclic Graphs (DAGs). See Rohrer (2018); Williams et al. (2018); and Wysocki et al. (2022).
3.	Avoid the “kitchen sink” method of covariate selection	Only include covariates in models after considering recommendations 1 & 2. Supplement 3 of Del Giudice and Gangestad (2021) is a useful resource to determine whether including a covariate is advantageous or deleterious, given the theoretical causal structure.
4.	Report standardized effect sizes	E.g., Cohen’s *d*, Pearson’s correlation coefficient, standardized β. See end of analytic code in supplementary materials for code to z-standardize variables to create a standardized β.

## Data Availability

Data and code are available in the Supplementary Materials.
