# Supplementary material for: Incorporating causal inference perspectives into psychoneuroimmunology: A simulation study highlighting concerns about controlling for adiposity in immunopsychiatry
Source: Brain Behav Immun. Author manuscript; Available in PMC 2024 Jul 5. (PMC11225100; doi:10.1016/j.bbi.2023.06.022)
Supplement: supplemental table 1 [file NIHMS2003214-supplement-supplemental_table_1.docx]

| Measure | 1. | 2. | 3. | 4. | 5. | 6. | 7. | 8. | 9. |
| --- | --- | --- | --- | --- | --- | --- | --- | --- | --- |
| 1. IL-6 | ‒— | .078** | .049* | .054* | .104** | .070** | .042 | .274** | .041 |
| 2. IL-8 | .078** | ‒— | .210** | .183** | .042 | .130** | .112** | -.078 | .034 |
| 3. IL-10 | .049* | .210** | ‒— | .101** | .170** | .021 | .025 | -.048 | .020 |
| 4. TNF-α | .054* | .183** | .101** | ‒— | .120** | .127** | .491** | .060 | .008 |
| 5. CRP | .104** | .042 | .170** | .120** | ‒— | .162** | .111* | .431** | .093** |
| 6. E-selectin | .070** | .130** | .021 | .127** | .162 | ‒— | .189** | .226** | .099** |
| 7. ICAM-1 | .042 | .112** | .025 | .491** | .11** | .189** | ‒— | .024 | .099** |
| 8. Adiposity | .274** | -.078 | -.048 | .060 | .431** | .226** | .024 | ‒— | .102* |
| 9. Depression  Symptoms | .041 | .034 | .020 | .008 | .093** | .099** | .073** | .102* | ‒— |

**Supplemental Table 1. Bivariate Correlations**

Note: IL- = Interleukin, CRP = C-reactive Protein, TNF-α = Tumor Necrosis Factor-α, ICAM-1 = Intracellular Adhesion Molecule-1
**p* < .05, ***p* < .01.
